# A rare case of primary congenital glaucoma in combination with neurofibromatosis 1: a case report

**DOI:** 10.1186/s12886-015-0142-8

**Published:** 2015-10-29

**Authors:** Haijun Li, Ting Liu, Xia Chen, Lin Xie

**Affiliations:** Department of Ophthalmology, Daping Hospital & Research Institute of Surgery, Third Military Medical University, Changjiang Branch Road, Chongqing, 400042 China

**Keywords:** Primary congenital glaucoma, Neurofibromatosis 1, Diagnosis

## Abstract

**Background:**

Neurofibromatosis 1 (NF1) is a common disease that mainly affects the skin and peripheral nervous system, and is characterized by bony dysplasia. Primary congenital glaucoma (PCG) is a sight-threatening disease that can manifest as a prodrome of NF1, especially in newborn babies. We report a case of PCG with NF 1.

**Case presentation:**

A 1-month-old boy presented with an enlarged right eyeball. An increased IOP and typical glaucomatous optic neuropathy were found, on the initial physical examination, a clinical diagnosis of primary congenital glaucoma (PCG) was made and a trabeculectomy with mitomycin C (MMC) therapy was subsequently performed. Three year later, the boy again presented with an even larger right eye and a gradually expanding left one. In addition to typical glaucomatous optic neuropathy, the boy also had multiple café au lait patches all over his body, megacephaly (head circumference = 60 cm; body weight = 14 kg; height = 93 cm) and remarkable facial features included swollen, soft upper eyelids and a flat, broad nose sphenoid wing dysplasia, eyelid thickening, bony orbit enlargement were found.

**Conclusions:**

It is rare have both PCG and NF1, and PCG may be a prelude to NF1. Continuous follow-up should be advised and we should raise our awareness of the combined condition and to improve chances for an early diagnosis.

## Background

Primary congenital glaucoma (PCG) is present at birth and is characterized by improper development of the eye’s aqueous outflow system. Increased intraocular pressure (IOP) results and subsequent damage to ocular structures and vision loss occurs. The prevalence of PCG around the world varies from 1 in 1250 to 1 in 22,000 [1], but its classic triad of symptoms (photophobia, epiphora, blepharospasm) may not be recognized until infancy or early childhood. Neurofibromatosis 1 (NF1), previously known as von Recklinghausen’s disease, is a common disease (birth incidence from 1 in 2500 to 1 in 4–5000) that mainly affects the skin and peripheral nervous system [2]. The skeletal system may also be affected and characteristic bony dysplasia can occur. Practitioners should keep in mind that all features of NF1 may not be present at birth. In 1987, the National Institutes of Health (NIH) Consensus Development Conference formulated the current diagnostic criteria and proposed the name neurofibromatosis 1 (NF1) [3]. A patient is considered to have NF1 if at least 2 of the following 7 criteria are met [4]:

- Six or more cafe’-au-lait macules with a diameter >5 mm in pre-pubescent individuals and >15 mm in post-pubescent individuals.
- Two or more neurofibromas of any type or one plexiform neurofibroma.
- Freckling in the axillary or inguinal regions.
- Optic nerve glioma.
- Two or more iris Lisch nodules.
- A distinctive osseous lesion (e.g., sphenoid wing dysplasia, long bone cortical thinning with or without pseudoarthrosis).
- A first-degree relative who meets the criteria for NF1.

In pediatric patients, it is often difficult to make an early NF1 diagnosis because several clinical manifestations appear as the child grows. This can be frustrating because children often have better outcomes with earlier intervention when subtle clues of NF1, such as congenital glaucoma, are recognized. Annual ophthalmologic evaluation, at least until the age of 7 years, can be a screening tool for increased IOP or other ocular disorders. This would help in the early detection of ocular disorders [3], including PCG, which can be the first symptom of NF1.

Age can influence tumor recurrence rates. Wise et al. demonstrated that surgical tumor resection before 10 years of age had a recurrence rate of 60 %, while resection performed on patients older than 10 years of age had a recurrence rate of only 30 % [5]. Additionally, Needle et al. showed that surgical intervention on the head, neck, or face before 10 years of age led to a shorter period of tumor control than intervention after age 10 [6]. Around 20 % of NFT-1 patients will develop a plexiform neurofibroma but only 1–2 % one will develop a malignant peripheral nerve sheath tumor (MPNST). NFT-1 and tumor volume have been widely identified as poor independent prognostic factors regarding tumor recurrence and patient survival. No reliable screening test exists in clinical practice yet, although recently potential markers for early diagnosis have been researched. Here, we report a case of PCG associated with NF1, which may provide awareness of these diseases and offer some insight on diagnosing them early.

## Case presentation

**Fig. 1:**
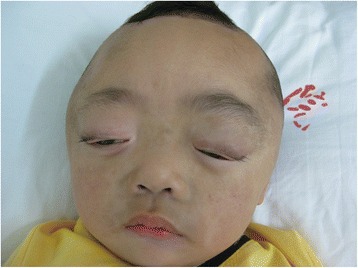
Droopy, swollen upper eyelids of both eyes and a flat, broad nose are apparent

**Fig. 2:**
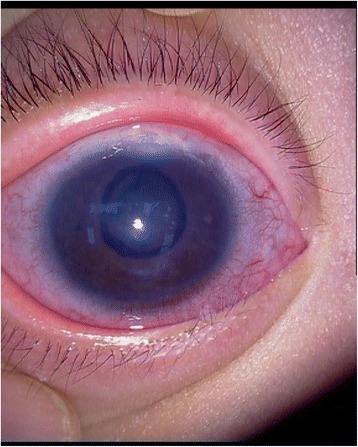
Corneal edema and Haab’s striae are readily apparent in right eye

**Fig. 3:**
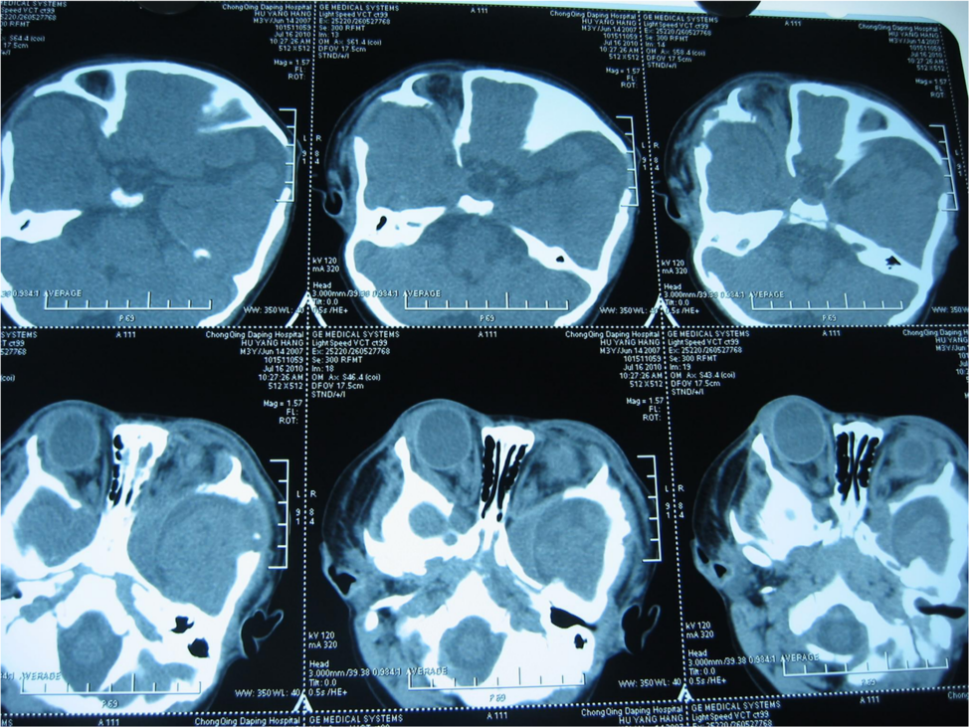
Focal areas of high signal intensity on T2 weighted MRI images in the left brainstem and the right cerebellum show likely gliosis or abnormal myelination. Dysplasia of the sphenoid wing is also present on the MRI of the head and orbit

**Fig. 4:**
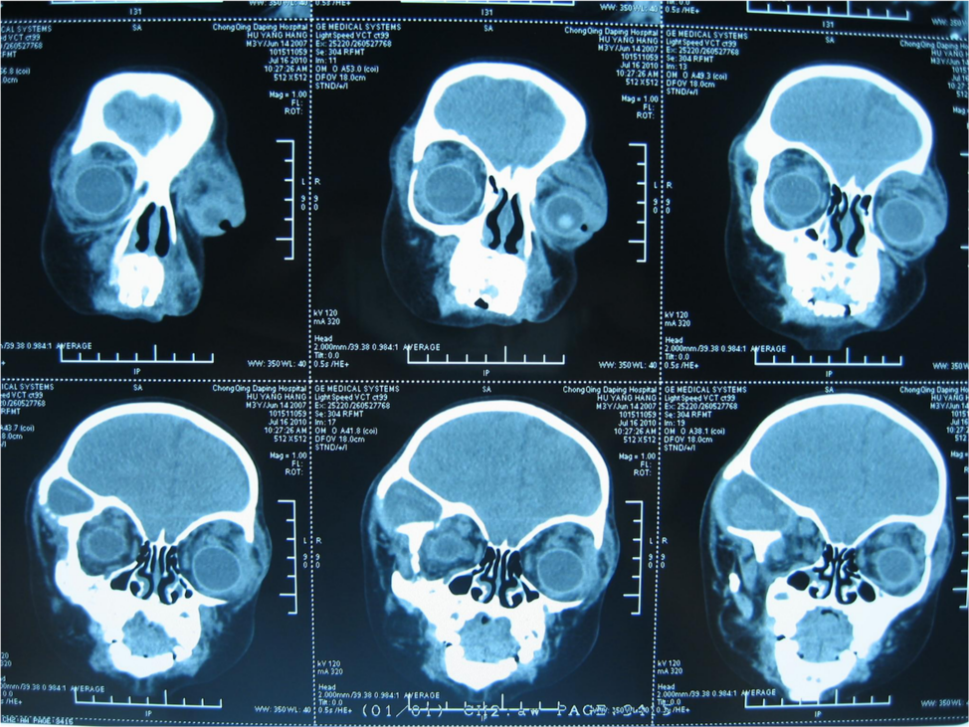
A coronal T2-weighted MRI shows focal areas of high signal intensity in the head and orbit. These may be the result of gliosis or abnormal myelination. And the sphenoid wing dysplasia is also present

**Fig. 5:**
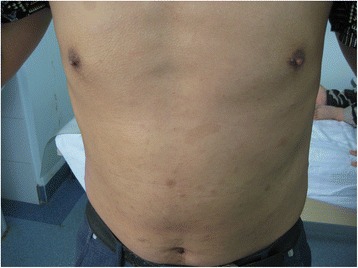
Multiple café au lait patches are apparent on the abdomen of the boy’s father

**Fig. 6:**
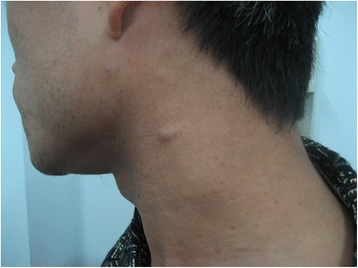
Non-tender, soft subcutaneous nodules are clearly visible on the left side of the father’s neck

A one-month-old boy presented to the outpatient department of our hospital with an enlarged right eyeball and persistent crying since birth in 2008. He was carried to term and delivered by Cesarean section to a first-time mother. The infant had no familial history of glaucoma or other ocular disorders. On initial physical examination, the right eye was notably larger than the left one and had a horizontal corneal diameter of 14 mm. Intraocular pressure (IOP) was 30 mmHg in the right eye and 14 mmHg in the left eye, as measured by a Tono-pen (Medtronic, Inc., Jacksonville, Florida). In the right eye, corneal edema and bullous keratopathy were present and fundoscopy revealed optic disc cupping and relatively healthy rim tissue. Orbit ultrasonography and brain computed tomography were unremarkable. On the basis of the above findings, a clinical diagnosis of primary congenital glaucoma (PCG) was made in the right eye and a trabeculectomy, with mitomycin C (MMC) therapy, was subsequently performed. The operation was successful but postoperative follow-up was limited because of parental non-compliance. Three year later, in 2011, the boy again presented to our clinic, but this time with an even larger right eye and a gradually expanding left one. Because the boy was also irritable and agitated, further examination was performed under anesthesia. Megacephaly (head circumference = 60 cm; body weight = 14 kg; height = 93 cm) was present and remarkable facial features included swollen, soft upper eyelids and a flat, broad nose (Fig. 1). Both corneal edema and Haab’s striae were apparent in the right eye (Fig. 2). The corneal diameter was 15.5 mm and IOP, as measured by a Tono-pen, was 35 mmHg. In the left eye, the corneal diameter was 12.5 mm and IOP was 14 mmHg. A failed filtering bleb, mydriasis, and a dissolved lens were apparent in the right eye and iris ectropin was present in both eyes. Lisch nodules were also present in both eyes, but were slightly more prevalent in the left. Cup-to-disk ratios were 1.0 and 0.6 in the right and left eyes, respectively, and blood vessels were shifted nasally in both eyes. Anterior chamber angles were wide open in both eyes, but abundant pigmentation was visible. Axial length was 31 mm in the right eye and 27 mm in the left eye, as measured by ultrasound examination. The boy also had multiple café au lait patches all over his body. Focal areas of high signal intensity on T2-weighted MRI images showed likely gliosis or abnormal myelination in the left brainstem and the right cerebellum. Sphenoid wing dysplasia was also clearly present (Figs. 3 and 4). Eyelid thickening, bony orbit enlargement, extensive orbital soft tissue infiltration, choroidal/scleral layer enhancement, and irregular nodule optic nerve sheath thickening were present and were thought to all be caused by orbital plexiform neurofibromas (PNF) of the posterior ciliary nerves surrounding the optic nerve. Multiple café au lait patches and non-tender, clear-cut, soft subcutaneous nodules were found on the boy’s father upon examination (Figs. 5 and 6).

Given the familial link, and the newly emerged macrocephaly, Lisch nodules, multiple café au lait patches, cerebral gliomas and cognitive impairment, a diagnosis of NF1 was made. An Ahmed valve was successfully implanted in the right eye to control IOP and post-operative IOP was 12–15 mmHg and remained well-controlled. The importance of continued ophthalmologic and neurological monitoring was stressed to the family before they were sent home.

## Conclusion

Our rare case is about PCG with NF 1 and PCG may be a prelude to NF1. And it is an example to us about the importance of continuous follow up. Also, we should raise our awareness of the combined condition and to improve chances for an early diagnosis.

## Discussion

We diagnosed a boy with PCG when he was first examined in 2008. A trabeculectomy, with MMC therapy, was subsequently performed. In the 3 years following this presentation, multiple NF1 features appeared, including macrocephaly, Lisch nodules, multiple café au lait patches, cerebral gliomas, and cognitive impairment. Therefore, we changed the boy’s diagnosis to a combination of PCG and NF1. In cases such as this, ophthalmologic and systemic explorations in the family can help determine the underlying cause of the congenital glaucoma. This is especially true in a newborn with unilateral glaucoma and orbital malformations, and the possibility of associated NF1 should be considered [7, 8].

Congenital glaucoma is usually managed surgically,. Ideally, patients will be less than 1 year old so that visual dysfunction can be minimized. When an NF1 -associated congenital glaucoma is diagnosed, visual loss due to ambiyopia and/or oncothlipsis is common and should be aggressively treated. Goniotomy or trabeculotomy are the preferred techniques for IOP control, but if they fail or cannot be performed, trabeculectomy, with or without supplemental therapies (i.e., adjunctive antifibrosis therapy, glaucoma drainage devices [GDDs], cyclodestructive procedures), can be considered. In our case, previous trabeculectomy failed 3 years after surgery, so an Ahmed glaucoma valve was implanted as an alternative technique.

Before neurofibromas in other systems induce malformation, dysfunction, and/or canceration, surgical resection is needed. It is known that NF1 patients have some canceration, and it has been reported that 10–20 % of neurofibromas become malignant tumors, most commonly peripheral nerve sheath tumors (MPNSTs) [9]. Rhabdomyosarcoma and/or brain tumors can also develop. Therefore, children with complex neurofibromas require lifelong management in a specialist multidisciplinary unit. Lastly, we suggest that NF1-associated congenital glaucoma should be treated in a multiple disciplinary setting, as early as possible. Unfortunately, analysis of the genetic mutation(s) responsible for the PCG and NF1 in this patient has not yet been performed and the responsible functional proteins have not yet been identified. Further studies on the molecular and genetic causes of this patient’s abnormalities are in the planning stages.

In conclusion, it is rare have both PCG and NF1, and PCG may be a prelude to NF1. Although similar cases have been previously described, it is important to present new reports that may provide clues to other practitioners. This could help allow earlier diagnoses of NF1 [10–12]. Our case can also be used as an example to parents about the importance of regular ophthalmological and neurological follow up.

## Consent

Written informed consent on behalf of the child was obtained from the child’s father for publication of this Case report and any accompanying images. A copy of the written consent is available for review by the Editor of this journal.

## Abbreviations

PCG: Primary congenital glaucoma
IOP: Increased intraocular pressure
NF1: Neurofibromatosis
